# Dissecting NMOSD pathogenesis through animal models: a mechanism-oriented systems perspective

**DOI:** 10.3389/fimmu.2026.1793855

**Published:** 2026-04-17

**Authors:** Siqi Qiu, Yingyu Zhang, Xiaoshuang Wang, Di Wang, Xuege Zang, Qiurong Yang, Yaxin Qv, Shuai Wang, Xuemei Han

**Affiliations:** Department of Neurology, China-Japan Union Hospital of Jilin University, Changchun, China

**Keywords:** animal models, aquaporin-4, neuromyelitis optica spectrum disorders, pathogenesis, translational medicine

## Abstract

Neuromyelitis optica spectrum disorder (NMOSD) constitutes a demyelinating condition of the central nervous system driven by autoimmune inflammation. A hallmark of its pathogenesis is the antibody-mediated injury of astrocytes, primarily targeting the water channel aquaporin-4 (AQP4). Animal models are indispensable for dissecting disease mechanisms and accelerating the development of new therapies. However, creating models that closely mirror NMOSD remains difficult, in part because immune tolerance limits the induction of the autoreactive responses central to the disease. This review follows NMOSD over time and surveys experimental systems across four interconnected mechanistic themes: breakdown of immune tolerance, T–B cell collaboration, antibody-mediated effector injury, and the formation of pro-inflammatory tissue milieus that sustain pathology. For each theme, we outline the rationale for model design, evaluate how well key pathological features are produced, and discuss the limitations that shape interpretation. Rather than offering a single continuous reconstruction of human NMOSD, these platforms capture complementary and only partially overlapping aspects of pathogenesis. Taken together, they provide a mechanism-oriented framework for understanding how upstream immune dysregulation, humoral immunity, and tissue-level permissive factors shape disease expression. At the same time, they also highlight the distance between experimental systems and the heterogeneous, dynamic course seen in patients. We further discuss how findings from animal studies are informing therapeutic target discovery and outline priorities for the development of next-generation models with greater translational relevance.

## 1 Introduction

**Figure 1:**
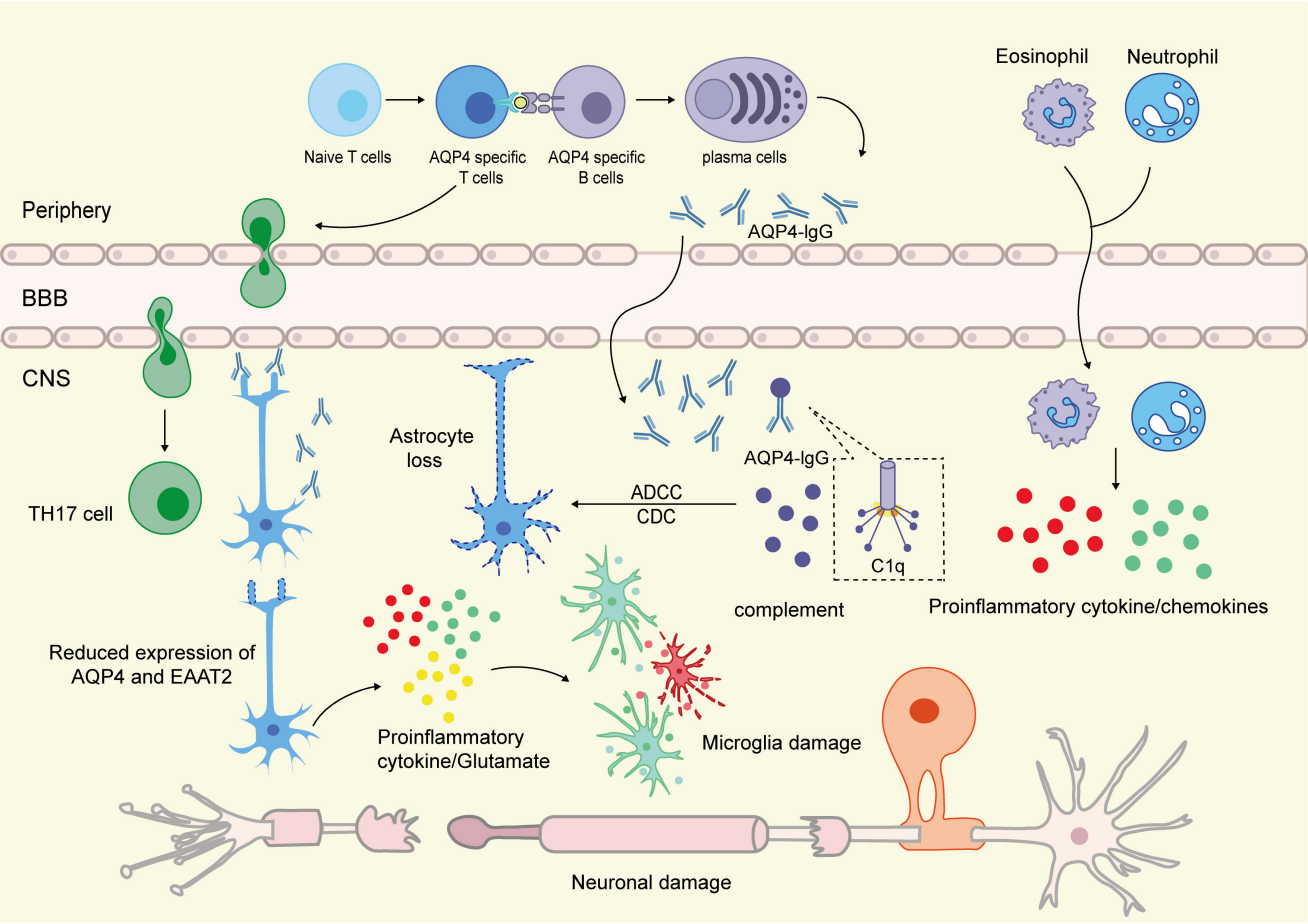
Pathophysiological mechanisms of NMOSD. AQP4-specific B cells, upon activation, can differentiate into plasma cells in the peripheral immune system and produce AQP4 antibodies. Under specific conditions, such as at anatomical weak points or in the presence of inflammatory mediators, these antibodies cross the blood-brain barrier (BBB) and enter the central nervous system, preferentially binding to the endfoot structures of astrocytes. This binding induces reactive changes in astrocytes, including altered expression of EAAT2 and AQP4, and may render them neurotoxic through the release of inflammatory cytokines and glutamate. Concurrently, activated antigen-specific T cells differentiate into Th17 cells following interactions with antigen-presenting cells. These Th17 cells not only cross the BBB but also promote increased BBB permeability, facilitating the infiltration of antibodies into the CNS and further recruiting inflammatory cells such as neutrophils and eosinophils. Within the localized inflammatory microenvironment, the complement component C1q binds to the antibodies, leading to astrocyte damage through complement-dependent cytotoxicity (CDC) and antibody-dependent cellular cytotoxicity (ADCC). This process is accompanied by the recruitment and infiltration of various immune cells, including neutrophils and eosinophils, into the lesion sites. Notably, the immune activation and damage mechanisms described above may further extend to neighboring oligodendrocytes and neurons via the “bystander effect,” thereby amplifying the scope of neural injury.

**Figure 2:**
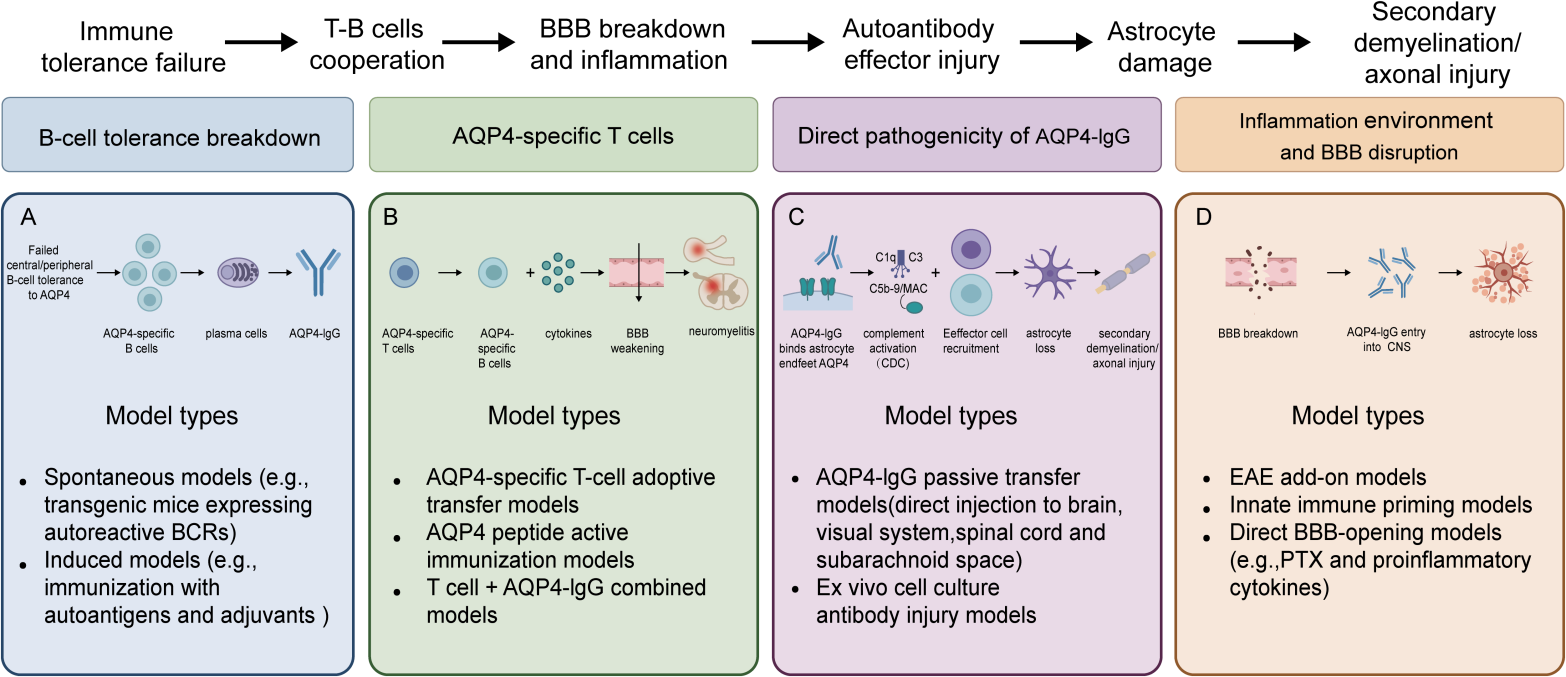
Pathogenic hypotheses and related model classifications. Based on researchers’ progressive understanding of the disease pathogenesis, the models can be broadly categorized into four major types: **(A)** models of B-cell tolerance breakdown, **(B)** models focusing on the helper function of AQP4-specific T cells, **(C)** models demonstrating the direct pathogenicity of AQP4-IgG, and **(D)** models based on inflammatory environments and blood-brain barrier disruption.

Neuromyelitis optica spectrum disorder (NMOSD) comprises autoimmune inflammatory diseases of the central nervous system (CNS). A central pathogenic hallmark is astrocyte injury mediated by autoantibodies targeting aquaporin-4 (AQP4) (1). NMOSD is conceptually an expanding spectrum. Around 80% of patients have pathogenic serum antibodies against aquaporin-4 (AQP4), and these cases correspond to “classical” neuromyelitis optica (2). The remaining ~20% are AQP4-IgG–negative or still of uncertain etiology, and it is this heterogeneous group that underpins the “spectrum” concept of NMOSD (3). Upon binding to AQP4 on astrocytic endfeet, AQP4-IgG can activate complement and promote the recruitment of immune effector cells, leading to astrocyte injury followed by secondary demyelination and axonal damage (4, 5). Histopathologically, NMOSD lesions differ from those of multiple sclerosis. They are characterized by marked loss of AQP4 at astrocytic endfeet, prominent perivascular deposition of immune complexes (IgG and complement), and inflammatory infiltrates enriched in neutrophils and eosinophils (6) (**Figure 1**). Despite the established pathogenic role of AQP4-IgG, the upstream cascade remains incompletely understood, including the loss of immune tolerance, the emergence of AQP4-specific autoantibody responses, and the basis for selective CNS vulnerability (7). Developing animal models that faithfully recapitulate human NMOSD has remained a longstanding challenge. Most hosts maintain strong immunological tolerance to the self-antigen AQP4, so conventional active-immunization strategies often fail to reproduce key disease phenotypes. This limits mechanistic investigation and reduces the predictive value of preclinical testing for candidate therapies. To address this obstacle, investigators have proposed four interrelated mechanistic frameworks and developed corresponding experimental systems, each designed to capture distinct disease-defining steps (8) (**Figure 2**). Because no animal model currently reproduces AQP4-IgG–negative NMOSD in a fully faithful manner, this review focuses on canonical AQP4-IgG–driven pathogenic mechanisms and the experimental models used to study them. To delineate a heuristic view from immune initiation to late-stage tissue injury, this review considers, in sequence (1): models of B-cell tolerance failure that probe the emergence of autoreactive immunity (2); approaches that emphasize the helper function of AQP4-specific T cells (3); passive-transfer paradigms that directly demonstrate the terminal pathogenicity of AQP4-IgG; and (4) combinatorial models showing that a permissive inflammatory milieu and blood–brain barrier (BBB) disruption facilitate lesion formation. Across these hypothesis-based categories, we compare the rationale for model construction, pathological phenotypes, mechanistic inferences, and inherent limitations. Importantly, we do not perform a single linear reconstruction of NMOSD pathogenesis, but rather utilize these models as a heuristic, mechanism-oriented framework to understand how different pathogenic processes interact under the constraints of experimental simplification.

## 2 B-cell tolerance breakdown: the origin of autoimmune responses and challenges in model development

This hypothesis addresses a central etiological question in NMOSD by positing that the initiating defect is an early breakdown of B-cell immune tolerance to the self-antigen AQP4 (9). Under physiological conditions, immature B cells expressing B-cell receptors that recognize self-antigens (e.g., AQP4) are typically subjected in the bone marrow or peripheral compartments to clonal deletion, receptor editing, or anergy, thereby establishing central and peripheral tolerance (10). In patients with NMOSD, however, defects at these tolerance checkpoints allow AQP4-specific B cells to escape control, become activated, and ultimately differentiate into plasma cells or memory B cells capable of producing pathogenic AQP4-IgG (9, 11). Clinical studies provide direct support for this concept: the proportion of naïve B cells in peripheral blood with the potential to generate AQP4 autoantibodies is increased in NMOSD patients, and these cells can differentiate into antibody-secreting cells *in vitro* without continuous antigenic stimulation, suggesting a preactivated phenotype or a state of tolerance escape (12).

**Figure 3:**
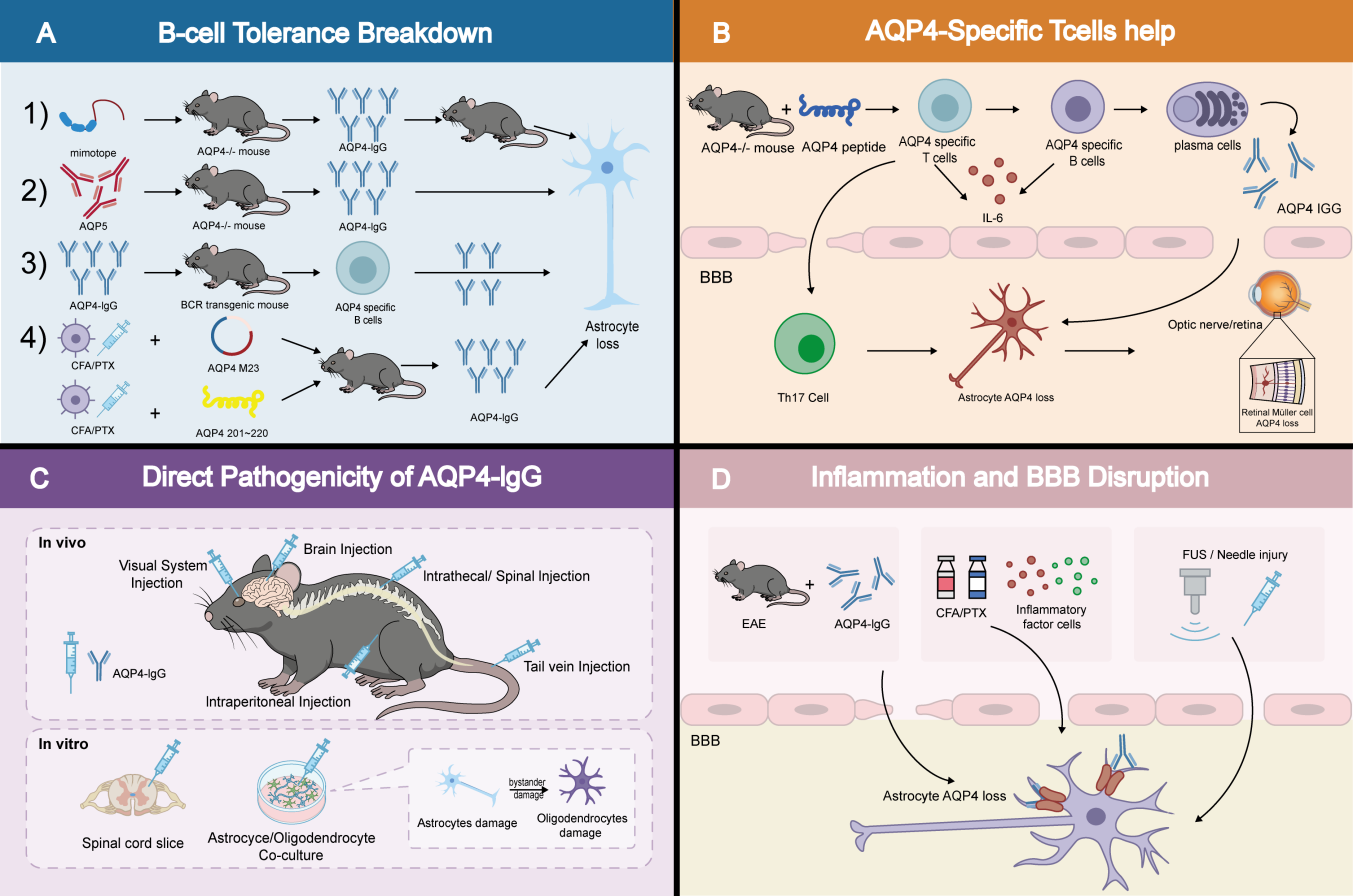
Schematic diagram of common animal model construction. **(A)** Model construction strategies related to B−cell tolerance breakdown; **(B)** Model construction strategies related to the helper function of AQP4−specific T cells; **(C)** Model construction strategies related to the direct pathogenicity of AQP4−IgG (*in vivo*/*in vitro*); **(D)** Model construction based on inflammatory milieu and blood−brain barrier disruption.

However, because wild-type animals exhibit strong tolerance to endogenous AQP4, conventional active immunization strategies rarely elicit a stable autoantibody response. To model the initial step of tolerance breakdown, investigators have developed a series of exploratory approaches. Epitope-mimic immunization is a prototypical strategy. For example, immunizing AQP4-knockout animals with mimotopes that mimic conformational AQP4 epitopes can induce high-titer AQP4-IgG. Subsequent passive transfer of these antibodies into wild-type animals triggers NMOSD-like astrocytic injury, supporting the pathogenic potential of antibodies generated via this route (13). Another approach leverages cross-reactive immunity. AQP4 belongs to the aquaporin family, and AQP0, AQP1, and AQP5 share partial sequence and/or structural homology with AQP4 (14). Immunization of AQP4-deficient mice with AQP5 has been reported to induce antibodies that cross-recognize AQP4 and, after AQP4 expression is restored, to provoke partial disease phenotypes such as optic neuritis (15). These findings further suggest that distinct autoimmune disorders may be linked through molecular mimicry. This provides a plausible explanation for the clinical comorbidity between NMOSD and conditions such as Sjögren’s syndrome (16). Recent studies have demonstrated that thymic B-cell subsets can upregulate endogenous AQP4 through CD40/IL-21-like signaling and present AQP4 to developing T cells, thereby promoting tolerance loss. When AQP4 expression is absent on B cells, the thymic tolerance checkpoint function is weakened, leading to the escape of AQP4-reactive clones from tolerance loss, which may facilitate the autoantibody response in NMOSD (17). Additionally, at a more mechanistically targeted level, B-cell receptor (BCR) transgenic models provide a means to directly track the *in vivo* fate of autoreactive B cells. Evidence indicates that thymic B cells contribute to the maintenance of central tolerance by expressing self-antigens (e.g., AQP4); BCR transgenic mice have been widely used to interrogate the fate of antigen-specific B cells and the mechanisms underlying tolerance breakdown (18, 19), demonstrating the feasibility of *in vivo* analyses of B-cell deletion, anergy, and autoimmunity driven by autoreactivity. In principle, introducing the heavy- and light-chain genes of a known pathogenic anti-AQP4 antibody into a BCR transgenic framework would enable animals to autonomously generate AQP4-specific B-cell clones, allowing direct assessment of whether these clones can breach tolerance during development and precipitate disease. Although such anti-AQP4 BCR transgenic models remain under active exploration, they hold promise as powerful tools for directly interrogating tolerance failure (**Figure 3A**).

Another intriguing approach is immunization-based modeling aimed at inducing endogenous anti-AQP4 antibody production. The underlying rationale is to “simulate” or “bypass” dysregulated tolerance mechanisms by presenting AQP4 in an artificially highly immunogenic form. For example, Yick et al. first perturbed the immune milieu with complete Freund’s adjuvant (CFA) and pertussis toxin (PTx), then electroporated a plasmid encoding the murine AQP4 M23 isoform into the tibialis anterior muscle to induce an immune response against self AQP4 under inflammatory conditions. AQP4-IgG was detectable in the sera of a subset of mice, accompanied by spinal cord inflammation and glial activation (20). Similarly, Serizawa and colleagues used multi-site subcutaneous immunization with AQP4_201_–_220_ plus CFA/PTx, inducing AQP4 autoantibodies, body weight loss, and spinal cord inflammation in C57BL/6J mice (21). Nevertheless, the reproducibility and phenotypic stability of these models remain to be further improved.

In summary, these exploratory models collectively demonstrate that immune tolerance to AQP4 can be disrupted and that autoantibody production can be induced in experimental animals, thereby providing valuable platforms for interrogating upstream immune events, including B-cell activation, the contribution of follicular helper T cells, and germinal center responses (20). In doing so, they shift the focus, at least in part, from downstream passive antibody effector mechanisms toward the origins of autoimmune responses. Nevertheless, substantial limitations remain. Most strategies still depend on strong exogenous immunostimulation, which makes it difficult to recapitulate the spontaneous, antigen-specific dysregulation of immune tolerance observed in human disease. Model performance is often unstable, with variability in antibody titers, pathological severity, and the consistency of lesion distribution. More critically, these models have not yet systematically reproduced the most characteristic features of NMOSD, including widespread AQP4 loss and the defining astrocytopathic phenotype. In patients with NMOSD, immune tolerance breakdown is likely multifactorial and may be promoted by genetic susceptibility, molecular mimicry, and environmental inflammatory cues acting on vulnerable immune checkpoints (9, 17, 22–24). Incorporating such upstream triggers into future model design may help bridge the gap between experimentally induced tolerance failure and spontaneous disease initiation. Consequently, the development of animal models that can more effectively capture early tolerance failure and its subsequent effects remains a core unresolved challenge in this field.

## 3 AQP4-specific T cells: a key helper model for disease initiation and immune amplification

The contribution of AQP4-specific T cells to NMOSD pathogenesis has become increasingly clear with advancing mechanistic understanding. In particular, helper Th17 cells (23, 25) not only provide essential signals to B cells to promote the generation of high-affinity, pathogenic AQP4-IgG (17), but also disrupt BBB integrity through the secretion of pro-inflammatory mediators and direct immune-cell infiltration toward specific CNS sites (e.g., the optic nerve and spinal cord) (26). In this way, they help establish the tissue selectivity of lesion formation. Accordingly, in the absence of a robust AQP4-specific T-cell response, it is difficult both to initiate the production of pathogenic antibodies and to enable their effective distribution and injury within the CNS.

To interrogate this T-cell–mediated role, multiple modeling strategies have been explored. Early efforts to establish models via adoptive transfer of AQP4-specific T cells were largely unsuccessful in fully recapitulating NMOSD (27). Although such approaches could elicit widespread inflammation and demyelination, they frequently failed to produce the characteristic, extensive loss of AQP4, potentially because these T cells preferentially localize to the meninges rather than broadly infiltrating the parenchyma (26). Arellano et al. immunized C57BL/6 mice with the AQP4_201_–_220_ peptide and found that concomitant disruption of IFN-γ signaling was associated with excessive IL-6 production and aberrant Th17 activation, culminating in severe NMOSD-like autoimmune pathology. These results highlight the pathogenic importance of Th17–B cell cooperation (7). Subsequent work further demonstrated that co-administration of such T cells with AQP4-IgG more reliably induced canonical pathological hallmarks, including BBB opening and complement deposition, indicating that T cells can create a necessary “permissive” milieu for antibody effector function (25, 26, 28). Notably, Zeka et al. (29) reported that T-cell immunization with specific AQP4 peptides can generate AQP4_268_–_285_–specific T cells; when these T cells were co-administered intraperitoneally with AQP4-IgG, they induced loss of AQP4 in retinal Müller cells and elicited partial clinical features such as optic neuritis and myelitis in recipient animals.

In addition, active immunization models aim to directly induce endogenous T-cell and antibody responses by immunizing with AQP4 antigens. Early studies showed that immunization of mice or rats with AQP4 protein or peptides could yield detectable antigen-specific T-cell proliferation and serum antibodies, yet failed to reproduce the clinical manifestations and core pathological features of NMOSD (30). More recent approaches have attempted to circumvent central immune tolerance by using T cells derived from AQP4-knockout mice, or by polarizing T cells *in vitro* toward a Th17 phenotype prior to adoptive transfer. These strategies successfully induced clinical features of myelitis and optic neuritis and generated demyelinating lesions in recipient mice; however, prominent AQP4 loss remained limited (28, 31) (**Figure 3B**).

T cell–based experimental models provide direct evidence for the presence of AQP4-specific T cells and their bias toward a Th17 phenotype. This is consistent with clinical observations showing increased Th17-related cytokines, including IL-6 and IL-21, in patients with NMOSD (31). To some extent, these models also reproduce the regional pattern of CNS inflammation and help explain the characteristic lesion distribution seen in NMOSD. Nonetheless, critical limitations persist. Without a strong humoral response, especially sustained production of high-titer pathogenic antibodies, T-cell immunization or adoptive transfer rarely reproduces a key feature of the extensive AQP4 loss (28, 31). This gap highlights the difficulty of capturing *in vivo* T–B cooperation and rebuilding the full immune sequence from antigen presentation to germinal center reactions and the emergence of high-affinity antibodies (12, 17, 32). In addition, Saadoun et al. showed that NMO patient IgG was not pathogenic when injected into mouse brain in the absence of compatible complement, whereas co-injection of NMO-IgG with human complement produced characteristic NMO-like lesions with loss of AQP4 and subsequent inflammatory demyelinating pathology (33). These results suggest that interspecific differences in complement activity may inhibit antibody effector function in T cell-dependent mouse systems. Mouse serum has low intrinsic classical complement activity, and contains potent inhibitors that target the classical complement pathway. Remarkably, even small amounts of mouse serum can strongly inhibit AQP4-IgG–dependent CDC mediated by complement from other species, thereby limiting the utility of mice for modeling complement-driven astrocyte injury (34). These constraints underscore a critical unmet need for models that more organically integrate cellular and humoral immunity and reliably reproduce the complete pathological spectrum of NMOSD.

## 4 Direct pathogenicity of AQP4-IgG: from passive transfer to mechanistic validation

The direct pathogenic role of AQP4-IgG is central to the mechanistic framework of NMOSD. Early studies suggested that AQP4 autoantibodies can drive tissue injury on their own: after binding AQP4 on the surface of astrocytes, they can activate the complement cascade and initiate downstream pathological changes (35). Histopathological studies have shown that many active NMOSD lesions exhibit loss of AQP4 accompanied by deposition of IgG and complement, supporting the important role of the AQP4-IgG-complement axis as a terminal effector pathway (36). In addition to ADCC/CDC, AQP4-IgG binding can also induce astrocyte dysfunction, such as mediating the endocytosis/internalization of AQP4 (37), and leading to impaired glutamate clearance through downregulation of the astrocyte glutamate transporter EAAT2 expression, resulting in functional imbalance (38). To directly test this hypothesis, investigators have developed multiple passive-transfer models in which AQP4-IgG and its effector molecules are delivered directly into the CNS of experimental animals—including intracerebral, intrathecal, intraspinal, and intra-visual system routes—to determine whether NMOSD-like astrocytopathic lesions can be induced.

For example, chronic intraventricular infusion of immunoglobulins containing patient-derived AQP4-IgG into the rat cerebrospinal fluid can induce widespread astrocytic injury across the brain and spinal cord, accompanied by reduced AQP4 expression, impaired glutamate uptake, and focal demyelination (39). Likewise, intravitreal injection of AQP4-IgG purified from patient serum into rats results in a marked decrease in AQP4 expression on retinal Müller glia, together with loss of ganglion cells and upregulation of glial fibrillary acidic protein (40). Notably, target-cell AQP4 internalization and degradation can still be observed after complement depletion with cobra venom factor, or when mutant AQP4-IgG lacking complement effector function is used. This indicates that, beyond the classical complement pathway, these antibodies can directly disrupt astrocyte function through endocytic mechanisms (40). *In vivo* imaging has further clarified the dynamics of antibody-induced injury. In a mouse spinal cord window model, administration of human AQP4-IgG together with complement causes rapid astrocyte loss within hours, followed by relative preservation of oligodendrocytes but progressive axonal swelling and injury (41). Collectively, these CNS-localized antibody delivery models provide direct support for the “direct pathogenicity of AQP4-IgG” concept: once AQP4-IgG gains access to the CNS and binds its target antigen, it is sufficient to initiate a contiguous pathological cascade centered on astrocytic injury.

Ex vivo experimental systems likewise support the direct cytotoxicity of AQP4-IgG. In astrocyte–oligodendrocyte co-cultures or organotypic spinal cord slice preparations, addition of AQP4-IgG together with complement induces selective astrocytic injury followed by secondary myelin loss (42). Moreover, Tianjiao and colleagues co-cultured AQP4-expressing cells with AQP4-negative cells and found that, upon exposure to AQP4-IgG and effector cells, not only were AQP4-positive cells killed, but neighboring AQP4-negative cells also exhibited “bystander” damage (43). The authors attributed this antibody-dependent bystander injury to directed release of cytotoxic granules by effector cells (e.g., NK cells and neutrophils) upon recognition of antibody-opsonized target cells, with collateral impact on nearby cells that are not bound by antibody. In addition, Bigotte et al. showed that purified AQP4-IgG from patients rapidly induces a specific reactive phenotype in rat ependymal cells (in primary cultures and ventricular wall explants), including AQP4 surface agglomeration, enlarged cell morphology, altered gap-junction features, impaired ciliary polarity/motility, and a pro-inflammatory transcriptomic response, suggesting ependyma as an early target potentially contributing to periependymal lesion formation (44). Together, these observations indicate that as a terminal pathogenic factor, AQP4-IgG can not only directly injure effector cells but may also broaden the extent of tissue damage through bystander mechanisms (**Figure 3C**).

These passive-transfer models successfully recapitulate the core histopathological hallmarks of NMOSD, including astrocyte loss, AQP4 depletion, IgG and complement deposition, and secondary demyelination, thereby providing direct evidence for the pathogenic sufficiency of AQP4-IgG. Consequently, these models are widely used to evaluate interventions targeting the terminal effector phase of NMOSD. In passive-transfer paradigms, complement inhibitors markedly reduce astrocytic injury and improve neurological outcomes (45–47). Several small-molecule agents, including minocycline and tanshinone IIA, have also shown therapeutic potential by mitigating neurological deficits (48, 49). Despite these strengths, passive-transfer models have inherent limitations. Lesions are typically acute in onset and spatially restricted to discrete CNS regions, limiting their ability to reproduce the relapsing–remitting clinical course of NMOSD and the multifocal, more diffuse tissue injury observed in patients. Many protocols rely on experimental manipulations to facilitate antibody transit across the BBB, whereas in human disease pathogenic IgG must gain CNS access from the peripheral circulation under physiological conditions. Taken together, these considerations indicate that, although passive transfer provides compelling evidence for the pathogenic capacity of AQP4-IgG, full manifestation of antibody effector mechanisms *in vivo* often requires concomitant alterations in the CNS immune microenvironment. This has provided a clear rationale for developing experimental models that more closely approximate the physiological disease process.

## 5 Inflammation and blood–brain barrier disruption: “permissive” environment models for antibody pathogenicity

Although AQP4-IgG is a central pathogenic autoantibody in NMOSD, seropositivity alone often fails to reproduce the full spectrum of NMOSD pathology. Accumulating clinical and experimental evidence suggests that effective engagement of antibody effector mechanisms typically requires a pre-existing inflammatory CNS microenvironment that provides a permissive context, often conceptualized as a “second hit” (8, 50, 51). Such a milieu can be generated by T cell–driven neuroinflammation (52) (e.g., experimental autoimmune encephalomyelitis), activation of innate immune pathways (53, 54) (e.g., lipopolysaccharide or complete Freund’s adjuvant), or direct physical/chemical compromise of the BBB (55) (e.g., ultrasound or needle puncture). In experimental animals, reinstating these conditions can substantially potentiate the CNS pathogenicity of AQP4-IgG.

Under physiological conditions, the intact BBB largely restricts the entry of circulating antibodies into the CNS, which may help explain why some individuals who are seropositive for AQP4-IgG remain asymptomatic for extended periods (56, 57). Animal studies indicate that, after intravenous administration, AQP4-IgG is distributed mainly to peripheral organs. Within the CNS, only minimal deposition is detectable, largely confined to regions that physiologically lack a BBB, and this occurs without evident tissue injury (58). Accordingly, disruption of the BBB is widely viewed as a pivotal prerequisite for enabling pathogenic antibodies to reach and engage CNS targets. Beyond barrier permeability per se, available evidence also suggests that an established inflammatory milieu can substantially potentiate antibody-mediated injury. Illustratively, Bennett JL and colleagues cloned and reconstructed a human anti-AQP4 monoclonal antibody from the cerebrospinal fluid of patients with early NMOSD and delivered it through the retrobulbar venous plexus to animals with experimental autoimmune encephalomyelitis (EAE), in which CNS inflammation was already in place. Under these conditions, they observed perivascular astrocyte loss accompanied by myelin disruption and complement deposition, consistent with key histopathological hallmarks of NMOSD (59). In a landmark study, Levy and colleagues first induced EAE in mice by active immunization with myelin oligodendrocyte glycoprotein amino acids 35–55 (MOG_35_–_55_), and then administered human AQP4-IgG by passive transfer. Compared with EAE-only controls, mice receiving AQP4-IgG developed more severe paralysis and formed larger and more extensive demyelinating lesions in the spinal cord (51). These lesions were predominantly located in superficial spinal cord regions enriched in AQP4, suggesting that during inflammation antibodies more readily penetrate the parenchyma and target astrocytes. Researchers have used EAE-inducing components to treat mouse brain endothelial cells *in vitro* and observed relocalization of the tight junction protein ZO-1, leading to increased permeability of the endothelial monolayer (60). This suggests that the EAE model is an ideal system for studying the disruption and repair of BBB tight junctions, as it provides a physical basis for changes in BBB permeability. Collectively, these findings indicate that the “subclinical inflammatory state” provided by EAE increases BBB permeability and creates the conditions that allow antibodies to enter the CNS and target astrocytes.

Beyond immune inflammation–mediated BBB disruption, direct physical or chemical opening of the barrier can also effectively “license” antibody pathogenicity. Asavapanumas and colleagues reported that following needle-prick injury to the mouse brain, intracerebral or intraperitoneal administration of AQP4-IgG was sufficient to generate typical lesions even in the absence of exogenously supplied complement or pro-inflammatory cytokines (61, 62). More targeted approaches, such as focused ultrasound (FUS) combined with microbubbles, can create a spatiotemporally controllable window of BBB opening in the brains of living animals. During this window, circulating AQP4-IgG can enter defined brain regions and induce characteristic pathology (55, 63, 64). Importantly, this process is strictly dependent on AQP4-IgG and its interaction with complement, thereby excluding nonspecific damage attributable to ultrasound itself. Additional interventions, including hyperosmotic mannitol (65) and pro-inflammatory cytokines such as IL-1β and TNF-α (66, 67), can similarly increase antibody access to the CNS through related mechanisms. However, we note that these BBB-opening methods are useful in experimental settings but are inherently artificial and may not fully reflect the initial barrier injury that occurs in patients. From this perspective, how peripherally generated AQP4-IgG gains access to the CNS during attacks becomes a key question. Most studies suggest that antibody entry may occur through transient and multifactorial barrier vulnerability. For example, in a human neurovascular-unit model, exposing AQP4-positive astrocytes to patient-derived AQP4-IgG induces IL-6 production, and IL-6 signaling to brain endothelial cells reduces barrier integrity and promotes leukocyte transmigration; this effect can be reversed by IL-6 neutralization (68). Consistent with this, a BBB-focused experimental study reported that IL-6 blockade suppressed BBB disorder and prevented disease onset (69). In addition to cytokines, humoral factors in NMOSD patient serum can induce human brain microvascular endothelial cells to secrete MMP-2/9 in an autocrine manner, which may increase BBB permeability (70), and it has also been reported that elevated MMP-9 levels are associated with markers of BBB disruption (71). Together, these observations provide hypotheses for the permissive “second hit” hypothesis and reveal a core feature of NMOSD attacks: peripherally generated antibodies typically exert their entire pathogenic effects only after BBB integrity has been compromised. At the same time, these paradigms offer tractable platforms for translational studies, enabling systematic evaluation of interventions that stabilize the barrier, dampen local inflammatory licensing signals, or interrupt antibody effector cascades within the CNS. For example, EAE add-on paradigms have been used to test the impact of certain multiple sclerosis therapies (e.g., interferon-β) in an NMOSD context, revealing potential exacerbation of disease, consistent with clinical observations and providing an important caution for therapeutic decision-making (64). In parallel, precision BBB-opening technologies such as FUS constitute enabling tool platforms for investigating targeted CNS drug-delivery strategies (72). MRI-guided FUS has been used to direct large monoclonal antibodies (e.g., trastuzumab/Herceptin) into specific brain regions in mice, and to enhance delivery and antitumor efficacy of liposomal doxorubicin within tumor areas in glioma models (73) (**Figure 3D**).

Nevertheless, these models also have important limitations. For instance, in EAE add-on paradigms, the intense T cell–driven inflammatory background elicited by myelin antigens (e.g., MOG) may not fully reflect the authentic triggers preceding disease onset in human NMOSD. The resulting pathological manifestations are a complex of NMOSD-like astrocytic damage and EAE-like demyelination, which complicates mechanistic interpretation. Meanwhile, pathological models capable of simulating BBB opening in patients are not yet mature. Therefore, a further objective is to develop more clinically realistic models that induce transient blood-brain barrier dysfunction or pathogenic innate immune activation without introducing heterologous highly immunogenic antigens, while acknowledging that such approaches still represent only selected components of the broader and more heterogeneous disease process observed in patients.

## 6 Translational value of NMOSD animal models

Animal models of NMOSD, based on different pathogenic hypotheses, should not be regarded as complete representations of a single disease course. Instead, they serve as complementary platforms for analyzing distinct pathogenic dimensions under specific conditions, including tolerance failure, T–B cooperation, antibody-mediated astrocyte injury, and inflammatory or barrier-dependent lesions. This makes them particularly valuable in preclinical studies linking mechanisms and therapies. In preclinical research on NMOSD, passive-transfer models are often prioritized for testing treatments targeting terminal antibody effector mechanisms, such as complement inhibition, Fc-effector blockade, or antigen decoy strategies, as they can recapitulate AQP4-IgG-mediated astrocyte pathology (59, 74, 75). In contrast, EAE add-on models or BBB-permissive models are more informative for interventions targeting inflammatory licensing, barrier disruption, or context-dependent lesion formation (51). Tolerance breakdown models and AQP4-specific T-cell models are better suited for investigating upstream immune initiation, T-B collaboration, and B-cell-directed immunomodulation (21, 28, 31). Therefore, no single model is universally preferred; rather, researchers typically select models that best align with the mechanistic profile of therapeutic candidates, with cross-platform validation enhancing translational advantages.

Passive-transfer studies provided early evidence that complement is not simply a secondary marker of injury, but a major effector pathway in NMOSD. Attenuating complement activity, or inhibiting proximal components such as C1q, substantially limits AQP4-IgG–dependent astrocyte damage and the associated inflammatory response (46, 61). These findings align with the prominent complement deposition seen in patient lesions (76). It also informed clinical strategies aimed at the terminal complement cascade, including C5 inhibition with eculizumab (77). Phase III trials subsequently reported clinical benefit, supporting its approval for AQP4-IgG–positive NMOSD. A similar translational logic applies to IL-6. Animal-model data implicating IL-6 signaling as a driver of disease amplification strengthened the rationale for IL-6 receptor blockade and informed the development of therapies such as tocilizumab and satralizumab (78). B-cell–directed strategies have also translated effectively. In the pivotal N-MOmentum trial, the anti-CD19 monoclonal antibody inebilizumab significantly reduced attack risk, with sustained benefit in long-term follow-up, highlighting the role of CD19 and B-lineage cells in relapse prevention (79, 80). Complement modulation can also be achieved upstream of C5 by leveraging endogenous membrane-bound regulatory factors. Notably, in astrocytic endfeet within the central nervous system, the expression of complement regulators such as CD55 and CD59 is relatively limited, and AQP4-expressing peripheral organs may therefore be more susceptible to AQP4-IgG–triggered complement-mediated injury (81). Studies have shown that restoring or upregulating these regulatory molecules (e.g., CD55) can attenuate complement-dependent pathology in NMOSD (82, 83). Importantly, these models also serve as the first *in vivo* proving ground for newer therapeutic concepts. For instance, Ding et al. used NMOSD mouse model to evaluate the effects of intravenous administration of the BAFF/APRIL-targeting fusion protein telitacicept. They reported that telitacicept reduced overall B-cell numbers and multiple B-cell subsets, lowered serum IgM, BLyS, and IL-6 levels, and downregulated transcription factors and receptors implicated in B-cell maturation and differentiation. Under experimental conditions, these changes reproduced immunological features consistent with a dampened humoral autoimmune response, in line with observations in patients (84). Meanwhile, complementary “antigen-decoy” approaches have also been investigated. Synthetic peptides corresponding to extracellular AQP4 epitopes have been used to neutralize AQP4-IgG in patient sera, thereby reducing antibody binding to astrocytes and limiting downstream effector protein–mediated injury. This strategy attenuated AQP4-IgG–driven cytotoxicity and conferred protection in animal models (85). Antigen-specific blockade strategies have also been developed. Aquaporumab exemplifies an antigen-specific “blocking antibody” approach for AQP4-IgG–positive NMOSD. It combines a high-affinity anti-AQP4 Fab with an Fc domain engineered to lack both complement activation and Fcγ receptor–mediated cytotoxic effector functions (74). By competitively occupying AQP4, Aquaporumab prevents binding of pathogenic patient IgG. *In vitro* and ex vivo studies reported near-complete suppression of patient-serum–mediated cytotoxicity, while *in vivo* experiments showed prevention of NMO-like lesions in mice, providing proof of concept for competitive inhibition at the initial antibody–antigen binding step (86). More recently, immunomodulatory and reparative strategies using human umbilical cord–derived mesenchymal stem cells (hUC-MSCs) have been explored. Intravenous infusion of hUC-MSCs improved motor performance and reduced inflammatory infiltration and demyelination in NMOSD mouse model, while preserving astrocytes and neurons and mitigating blood–brain barrier disruption. *In vitro*, hUC-MSCs further increased astrocyte viability and decreased apoptosis under AQP4-IgG and complement-mediated injury conditions, providing supportive preclinical evidence for the translational development of cell-based therapy (87). In addition, therapeutic strategies that are not yet established for NMOSD but are being actively developed for multiple sclerosis—such as Bruton’s tyrosine kinase (BTK) inhibitors (88) —have shown potential relevance. BTK signaling has been implicated in B-cell activation and myeloid responses in NMOSD, and BTK inhibition has demonstrated preclinical benefit in experimental NMO models (89). We also note that certain agents, such as interferon-β (IFN-β), are effective in multiple sclerosis largely by activating the IFNAR–STAT pathway, suppressing pro-inflammatory Th1/Th17 programs, and reducing antigen presentation and inflammatory cell activation. In parallel, IFN-β downregulates adhesion molecules and matrix metalloproteinases (e.g., VLA-4/VCAM-related adhesion and MMP-9), thereby limiting lymphocyte trafficking across the BBB into the CNS and exerting anti-inflammatory effects (90). In NMOSD, however, type I interferon signaling may instead synergize with the IL-6–B cell–Th17 axis, creating an amplifying loop. Agasing and colleagues identified an IFN-high subgroup in NMOSD and proposed that IFN-I promotes pathogenic Th17 differentiation by inducing IL-6 production in B cells; consistent with this, in Th17-driven EAE, IFN-β can upregulate IL-6 and exacerbate disease (91). Together, these observations underscore that NMOSD and multiple sclerosis, despite superficial overlap, diverge fundamentally in therapeutic responsiveness, and highlight the need for clinical caution when extrapolating therapies across the two conditions.

Looking forward, NMOSD animal models will remain an important bridge between mechanistic insight and therapeutic development. They enable evaluation of candidate interventions while linking treatment effects to specific pathways. As models become more human-relevant—for example, through improved Fc/complement compatibility and more faithful representation of BBB vulnerability—they should strengthen preclinical decision-making and reduce the likelihood of advancing ineffective or unsafe strategies into clinical trials. Beyond pharmacological approaches, it is plausible that animal models could be adapted to test gene-editing strategies aimed at the earliest pathogenic steps—for example, CRISPR-based interventions to remove or inactivate autoreactive B-cell receptor genes—thereby shifting prevention closer to the source of disease.

## 7 Limitations and future perspectives

A central limitation of current NMOSD modeling is that no single experimental system captures the full temporal, spatial, and immunological complexity of human disease. Passive-transfer models directly demonstrate the pathogenic sufficiency of AQP4-IgG and are widely regarded as the “gold standard” for interrogating downstream antibody effector mechanisms, yet they typically model acute, focal injury and therefore have limited capacity to recapitulate the chronic relapsing course of the disease (92). EAE add-on models introduce T cell–mediated inflammation and thus better approximate attack-like settings, but their readouts are confounded by anti-myelin immunity, complicating mechanistic dissection of astrocyte-specific injury (93). By contrast, active immunization models and tolerance-breakdown models target upstream initiation: the former enables longitudinal tracking of the activation and evolution of AQP4-specific T and B cells (32), whereas the latter directly models impaired immune tolerance as a primary defect (94). Although these approaches can be collectively regarded as highlighting the primary mechanistic dimensions of NMOSD, they should not be interpreted as additive proofs of a single unified pathological sequence. In reality, disease progression may exhibit nonlinearity and heterogeneity among patients, with variations in immune triggers, lesion localization, timing of attack, and downstream tissue responses. Consequently, future model development may no longer rely on a single “ideal” unified model but rather on constructing modular, human-relevant platforms tailored to specific biological questions, while enhancing the ability to examine interactions between upstream immunity, barrier fragility, and tissue damage. One plausible route is to generate transgenic animals co-expressing AQP4-specific T-cell receptors (TCRs) and B-cell receptors (BCRs). The MOG-specific BCR/TCR double-transgenic mouse model is relatively well established (95), while not reproducing full NMO pathology, shows NMO-like changes and is useful for defining autoreactive T–B cooperation (50). By analogy, establishing transgenic animals co-expressing AQP4-specific BCRs and TCRs would help elucidate the mechanisms underlying immune-mediated demyelination.

Despite considerable progress in animal modeling of NMOSD, significant gaps persist in how accurately current systems reflect the complexity of the human disease. A major gap is that many rodent paradigms do not naturally reproduce the characteristic anatomical predilection of human NMOSD (optic nerve, spinal cord, periependymal/periventricular regions, and area postrema) (49). Human pathology indicates that lesion distribution tracks regions of high AQP4 expression and specialized astrocyte architecture, including periventricular/hypothalamic regions and the medullary floor/area postrema (96). However, this phenomenon can hardly be simulated accurately in rodents. Future models may develop designs that specifically target multiple brain regions, where antibody attacks can better simulate the clinical symptoms of NMOSD, aiding in the understanding of its pathological mechanisms. Furthermore, the current repertoire of NMOSD animal models remains limited in its ability to capture disease dynamics. Most paradigms are designed to interrogate acute effector injury and therefore do not reliably reproduce the hallmark relapsing–remitting course of NMOSD. This limitation is partly technical. In passive-transfer paradigms, repeated administration of exogenous human IgG can frequently trigger serum sickness and other type III hypersensitivity reactions (86, 93). These complications undermine experimental durability and constrain long-term study designs. In active-immunization models, disease manifestations typically develop within weeks after immunization and seldom display clearly demarcated inter-attack intervals (21). Accordingly, the development of models capable of reproducing relapse and long-term chronic demyelination has become one of the current hotspots in model construction.

In addition, current evaluation frameworks tend to emphasize histopathological endpoints and motor impairment, while giving limited attention to non-motor manifestations that are common in NMOSD and can substantially reduce quality of life. Chronic neuropathic pain is reported in 72–86% of patients, and more than one third develop comorbid affective disorders such as depression and anxiety; these symptoms may persist even during clinical remission (97, 98). Area postrema syndrome is another hallmark domain. It is characterized by intractable nausea, vomiting, and/or hiccups with dorsal medullary involvement, and it can precede or occur independently of optic neuritis and myelitis (99, 100). Visual dysfunction is likewise central to disability in NMOSD, yet many preclinical studies still lack standardized functional visual evaluation criteria, despite the availability of AQP4-IgG based optic neuritis models (101, 102). While preliminary evidence suggests that AQP4-IgG may promote nociceptive sensitization through glia–neuron interactions (103), few studies systematically incorporate pain-related assays (e.g., mechanical and thermal threshold testing) together with depression-like paradigms (e.g., the forced swim test and sucrose preference test). However, surrogate readouts—such as pica (104) and conditioned taste aversion—together with dorsal medulla–focused pathology and glial activation markers can better approximate the clinical phenotype. For vision, optokinetic tracking, visual evoked potentials, OCT/retinal measures, and retinal ganglion cell quantification can be integrated with optic nerve pathology to yield clinically relevant endpoints (101). Future work should prioritize multi-domain neurobehavioral assessment and develop models that capture these symptom domains. This will help link immune attacks to chronic pain, affective dysfunction, brainstem symptomatology, and visual loss, and provide a stronger preclinical basis for therapies targeting these disabling features.

Species-related limitations in current model systems deserve careful attention. Most studies rely on rodents, but rodent immune biology, BBB structure and function, and complement activity differ from humans (105). This can reduce confidence in efficacy and safety predictions, especially for humanized monoclonal antibodies. In mice, classical complement effector function is often a key constraint. Mouse serum contains inhibitors that can strongly suppress AQP4-IgG–dependent CDC, even when complement from other species is added. This likely explains why many passive-transfer studies require exogenous human complement or other workarounds to generate NMO-like pathology (33, 34). Rat systems are often more permissive for human IgG–driven complement and Fc-mediated injury. Developing highly humanized mouse platforms (e.g., carrying human immune cells and/or human AQP4 epitopes) represents an important avenue to enhance translational value. PBMC-engrafted anti-NMDAR encephalitis models illustrate what such approaches can achieve: they produce patient-relevant immune activity together with BBB and inflammatory changes that allow *in vivo* mechanistic testing (106). Similar strategies have been used in myasthenia gravis, including SCID–patient lymphocyte chimeras and mice engineered to produce human antibodies, enabling *in vivo* testing of pathogenic human monoclonals (107, 108). Meanwhile, current NMOSD models still underrepresent clinical heterogeneity and common comorbid autoimmunity, such as systemic lupus erythematosus and Sjögren’s syndrome (109, 110). Composite models that combine multiple autoimmune responses may help clarify mechanisms of comorbidity and interactions between immune programs.

The fundamental limitation of current NMOSD animal models lies in their limited capacity to capture spontaneous autoimmune initiation. This makes it challenging to elucidate the mechanisms underlying the emergence of AQP4-directed immunity and the variations in clinical phenotypes among different patients. In many experimental paradigms, AQP4-IgG is exogenously administered and disease induction relies on potent adjuvants and BBB dysfunction. While these approaches are valuable for studying downstream effector-mediated damage, they provide limited insights into upstream susceptibility. The initiation of disease is likely multifactorial, potentially involving genetic background, molecular mimicry, and environmental or dietary-related immune dysregulation. Patient data indicate that NMOSD is significantly associated with HLA susceptibility, with enrichment of the DRB1*03 allele group in patients (111). Across populations, DRB1*03:01 has been repeatedly implicated in Western cohorts (112), whereas DPB1*05:01 and other haplotypes are more frequent in Asian cohorts (113). This points to a population- and region-dependent immunogenetic background. Furthermore, molecular mimicry provides a plausible explanation for the microbial and AQP4-directed autoimmune responses in NMOSD. One study showed that AQP4-specific CD4+ T cells from patients display Th17 skewing and cross-react with a homologous epitope from a *Clostridium perfringens* ATP-binding cassette (ABC) transporter permease (23). Consistent with this, the gut microbiome analysis revealed enrichment of Clostridium perfringens in NMO (114). Experimental data further suggest cross-immunoreactivity between bacterial aquaporins (e.g., AqpZ) and human AQP4, supporting the idea that infection-related antigens may prime AQP4-specific autoimmunity (115). Modifiable environmental factors may influence susceptibility to NMOSD, including reduced vitamin D levels (116) and diet-related inflammatory dysregulation. Observational studies using dietary inflammatory markers (117), high-fat diets (118), and sugar intake (119) have reported associations with NMOSD prevalence. Mechanistic studies suggest that LDL can enter the CNS when the BBB is compromised, activating microglia and exacerbating demyelinating damage (120). This multifactorial pathogenic system increases the difficulty of constructing animal models. Integrating these factors into model design may help correlate immune initiation with clinically patterned lesion distribution and recurrence propensity, better elucidate the upstream states of NMOSD disease, and enhance the clinical translational predictive capacity of the model.

## 8 Conclusions

Current NMOSD animal models collectively reveal multiple major pathogenic dimensions of the disease, ranging from immune tolerance disruption, autoreactive T–B cell interactions, antibody-mediated astrocyte damage, to blood-brain barrier dysfunction and the enabling role of local inflammatory environments. However, these models fail to simulate the true, continuous disease progression in humans. Instead, each model captures only partial components of the dynamically evolving disease course. Significant limitations remain in constructing models with spontaneous onset, relapsing–remitting dynamics, anatomical selectivity, and multidimensional clinical phenotypes (including pain, mood disorders, brainstem symptoms, and visual dysfunction). Species-specific differences in complement activity and immune responses, coupled with the multifactorial pathogenesis of NMOSD, further constrain translational predictability. Therefore, future research priorities should not only focus on enhancing model integration and clinical relevance but also recognize the potential need for distinct experimental models to address different mechanistic and therapeutic questions. With continuous improvements in animal models, this field will yield better mechanistic insights, preventive strategies, and interventions.
